# Putting Physical Activity Where It Fits in the School Day: Preliminary Results of the ABC (Activity Bursts in the Classroom) for Fitness Program

**Published:** 2010-06-15

**Authors:** David L. Katz, Daniel Cushman, Jesse Reynolds, Valentine Njike, Judith A. Treu, Catherine Katz, Jennifer Walker, Erica Smith

**Affiliations:** Yale-Griffin Prevention Research Center; Yale University School of Medicine, Derby, Connecticut; Yale University School of Medicine, Derby, Connecticut; Yale University School of Medicine, Derby, Connecticut; Yale University School of Medicine, Derby, Connecticut; Yale University School of Medicine, Derby, Connecticut; Independence School District, Missouri; Independence School District, Missouri

## Abstract

**Introduction:**

Despite well-documented evidence that physical activity is beneficial to children, average fitness levels of US children have declined. Lack of physical activity has been associated with childhood obesity. We evaluated the effects of a physical activity program in the elementary school classroom on health outcomes.

**Methods:**

Three schools in the Independence School District in Independence, Missouri, were assigned to receive the ABC (Activity Bursts in the Classroom) for Fitness program, and 2 comparable schools served as controls. The program, led by classroom teachers, provides multiple, brief, structured physical activity breaks throughout the day. Baseline data for the study were collected in September 2007, and follow-up data were collected in April 2008.

**Results:**

Physical fitness measures of upper-body strength, abdominal strength, and trunk extensor improved (*P* <.001). Medication use for asthma (*P* = .03), attention-deficit hyperactivity disorder (*P* = .07), or either medication combined (*P* = .005) decreased.

**Conclusion:**

The effects of the program on daily physical activity, fitness, and measures of health are beneficial.

## Introduction

The US Department of Health and Human Services (HHS) recommends that children and adolescents engage in 60 minutes of physical activity per day (1). HHS indicates that meeting this recommendation can improve cardiorespiratory fitness, muscular strength, blood pressure, and can decrease depressive symptoms in children in a short amount of time (1). Other studies have also concluded that physical activity in children and adolescents may be positively correlated with academic achievement (2).

Although the health benefits of physical activity for children are well documented, the average fitness levels in children in the United States have been declining. This may be due in part to declining levels of daily physical activity in the school setting and outside of it (3-6). Obesity raises the risk for type 2 diabetes mellitus, insulin resistance, heart disease, high blood pressure, metabolic syndrome, and other health-related disorders. Type 2 diabetes, once uncommon in childhood, now represents 8% to 45% of all diabetes reported in children and adolescents (7).

A nationwide survey found that parents seek ways to combat childhood obesity from schools more often than from health care providers and government agencies (8). No other institution has as much continuous and intensive contact with children (9). Schools can help combat obesity by offering physical education programs and recess, healthy school meals and foods, health education, and school health services.

Some experts have argued that meeting the academic requirements of the No Child Left Behind Act has forced US school districts around the country to choose between physical activity and academic activity (10). Existing school-based nutrition and physical activity programs are often resource- and time-intensive and compete with teaching. More school-based health-promotion programs are needed that enable schools to fulfill their primary academic mission (11). In this study, we evaluated the effects of a classroom-based physical activity program on physical fitness, academic performance, classroom behavior, and health outcomes of elementary school students. We hypothesized that including this program in elementary schools would improve the children's physical fitness, academic performance, classroom behavior, and health outcomes.

## Methods

The Independence School District (ISD) in Independence, Missouri, collaborated with Yale University's Prevention Research Center to implement and study a nutrition and physical activity program for elementary-school children. The study protocol and consent form were approved by the Griffin Hospital (Derby, Connecticut) institutional review board and the Yale University (New Haven, Connecticut) human subjects committee. The project aimed to promote a healthy lifestyle and reverse the trend of obesity among students and their families. The nutrition intervention has been described separately (12). The physical activity component, ABC (Activity Bursts in the Classroom) for Fitness incorporates brief bursts of activity in the classroom throughout the day at the discretion of the teacher. The project also features parental education and community involvement. We focused on providing a low-cost and sustainable intervention for classroom teachers to integrate into the existing school structure.

Developed with input from experts in education, the ABC for Fitness program encourages teaching during the activity bursts. ABC for Fitness aims to provide fun and creative activities that are noncompetitive, age-appropriate, and gender-neutral to promote an interest in physical activity among children and increase their behavioral capacity. It was designed to take the time that teachers spend getting restless students to settle down, or distracted students to concentrate, and convert this time into structured, productive bursts of physical activity spread throughout the day. The bursts were conducted during "down time" in the school day to help increase total daily teaching time. The program was flexible; activity intensity could be raised or lowered according to the athletic abilities and attention spans of the students. The program was intended as a supplement to physical education programs. Ideally, the activity bursts added at least 30 minutes of daily physical activity.

Each activity burst had 3 components: 1) a warm-up that could include stretching or light aerobic activity (eg, walking, arm circles, muscle stretching), 2) a core activity consisting of strength activities or aerobic activities (eg, hopscotch, lunges, squats, star jumps, jogging, walking quickly, hopping, dancing to music, skipping), and 3) a cool-down similar to warm-up activities, consisting of stretching or low-intensity activity. Although teachers were instructed to use all 3 components, they were not required to offer a particular ratio of strength activities to aerobic activities.

The length and the daily number of activity bursts could vary. Several sample activities, class configurations, and educator resources were in the ABC for Fitness teacher's manual (13). Teachers could select different options for each warm-up, core activity, and cool-down. They also chose from options to meet their classroom needs, including 1) "basic activity bursts" to provide a break between classes, meet students' need to move periodically, and help them channel their energy so they can refocus on learning; 2) "advanced activity bursts" that combine sets of movements into engaging classroom activities and contribute to overall fitness; 3) "activity bursts of imagination" that use creativity to move in the classroom; and 4) "activity bursts for learning and fitness" to facilitate hands-on learning in language arts, social studies, music, mathematics, science, and health classes.

### Subjects

For this study, 1,216 students from grades 2 through 4 in ISD were asked to participate. Two students dropped out of the study because of lack of parental consent. The ISD student population is predominantly white and has few minority students. Overall, 62% of the student population is eligible for free and reduced-price meals. Eligibility rates approach 95% in some schools (14), and nearly half of the children belong to households receiving food stamp assistance.

The Missouri Department of Elementary and Secondary Education reported that 49% of girls and 37% of boys failed to meet aerobic capacity requirements of the Missouri Physical Fitness Assessment in 2004 (15), a 10% decline for girls and a 2% decline for boys from 2000 state assessment data. Physical activity levels in abdominal and upper-body strength and flexibility show a 9% to 14% decline from 2000 figures (15). In a 3-year trend report by the Independence Health Department, 28% of ISD adolescents in 2005 reported that they exercised for 20 minutes up to 2 times per week, a 19% decline from 47% of these adolescents in 2003 (16). The sedentary lifestyle is widespread; more than 55% of ISD students report watching television 2 or more hours every school day, compared with 38% of students reported nationally in the 2003 Youth Risk Behavior Survey (17).

### Protocol

The implementation of the ABC for Fitness program began in August 2007 when training sessions were provided for classroom and physical education teachers before the start of the school year. Teachers completed a pretraining and post-training survey to assess changes in outcomes such as level of exercise literacy, general knowledge regarding physical activity, and self-efficacy for program implementation. The purpose was to establish the efficacy of the intervention in a randomized controlled trial. Five schools were randomly assigned to receive the intervention or to serve as the control group. The intervention also included a family/parental component in which fitness experts helped families learn how to be more active together. Schools assigned to the control group continued normal curricular activities during the intervention period and implemented the program in the following academic school year.  The inclusion criterion for the study was parental consent for students in both the intervention and control schools.

Parents were sent an information sheet describing the study and were asked to contact the principal of their child's school if they chose to have their child opt out of participating in the study. Exclusion from the study was the result of either a lack of parental consent (in written form) or the inability of the child to participate in the physical activity program because of physical limitations. Baseline data were collected in September 2007 and follow-up data were collected in April 2008.

### Outcome measures

Height and weight were measured for each child by the school nurse or wellness coordinator during a specified time set by school administrators. Children were measured fully clothed, except for shoes, and were not required to fast. A computerized body mass index (BMI) assessment tool, BMIforKIDz (GrowthCharts4Kidz, Ltd, Dublin, Ireland) was used to measure and record student BMI.

Assessment of endurance, strength, and flexibility was collected by Fitnessgram (18) in the fall and spring of each school year (October/November and April/May). Aerobic capacity was measured by The Pacer, a 15- or 20-meter progressive, multistage shuttle run set to music. Maximal oxygen consumption (VO_2max_) was measured as a proxy for general fitness by using the recommended calculation based on the number of laps completed and child's age (18). Abdominal strength was measured by curl-ups, upper-body strength by 90-degree push-ups, back extensor strength by the trunk lift, and flexibility by the back-saver sit and reach.

Classroom behavior was assessed by the work and social skills component of the ISD progress report for the 2007-2008 school year. This information is compiled on students quarterly. It comprises 14 classroom behavior-specific items that are rated on a 3-point scale in which 3 is the most desirable score (satisfactory) and 1 is the least desirable (needs improvement). The average score of these 14 items for the first (December 2007) and last quarter (June 2008) was used to assess behavior before and after the intervention for students in the second, third, and fourth grades.

Student use of medication for attention-deficit hyperactivity disorder (ADHD) and asthma were collected by school nurses at each school at the beginning and end of the intervention period. These data were collected using a standardized medication log. No encouragement, suggestion, or guarantee of reduced use of medication for asthma or ADHD in children was given to parents at the outset of the study.

Student attitudes toward physical activity were measured before and after the intervention by using a subset of the School Physical Activity and Nutrition (SPAN) Questionnaire. SPAN is a validated questionnaire (19). Questionnaire items inquired specifically about physical activity. Parents of students were asked to oversee the completion of the questionnaire with their child. These 6 questionnaire items were then summed to form a total score; the highest value (ie, 9) was the most desirable.

Academic performance was assessed by comparing postintervention Missouri Academic Performance (MAP) scores of fourth-grade students with their preintervention MAP scores from third grade (2006-2007 school year). Classroom grades from the communication arts and mathematics components of the ISD Progress Report for the 2007-2008 school year for students in the second, third, and fourth grades for the first (December 2007) and last (June 2008) quarterly marking periods were also used to assess academic performance.

### Data analysis

To assess groups' differences at baseline between the intervention and control groups, *t* tests, Pearson's χ^2^ tests, and Mann-Whitney tests were used. Repeated-measures analysis of variance was used to assess the between-group differences for the anthropometric and physical activity measures. Pearson's χ^2^ was used to assess between-group differences for physical activity, medication use, and both academic performance measures. Wilcoxon rank sum test was used to assess between-group differences in anthropometric and physical fitness measures. The effect of school (site) on outcome measures was assessed by using repeated measures and logistic regression. All analyses were based on intent-to-treat principle with the exception of the analysis physical activity measures, which had a poor response rate at follow-up. The statistical software package SPSS version 15 (IBM, Chicago, Illinois) was used to conduct all analyses. All tests were conducted with an α level set at .05.

## Results

A total of 1,214 students initially enrolled in the study — 655 students in the intervention group and 559 students in the control group. The same cohort of students in each group was measured at follow-up; however, the response rates varied on outcome measures (Figure). The 2 study groups were similar in terms of demographic characteristics: sex (*P* = .49), grade level (*P* = .98), and age (*P* = .89), anthropometric measures: BMI (*P* = .06), BMI percentile (*P* = .21); use of medication for asthma (*P* = .44) or ADHD (*P* = .47) or both (*P* = .29); and academic performance: MAP mathematics (*P* = .14) and communication arts (*P* = .22), ISD progress report mathematics (*P* = .08) and communication arts (*P* = .48)  (Table 1). The study groups were not similar at baseline in terms of weight (*P* = .004), aerobic capacity (*P* <.001), abdominal strength (*P* <.001), upper-body strength (*P* <.001), trunk extensor (*P* <.001), left-side flexibility (*P* <.001), right-side flexibility (*P* <.001), classroom behavior (*P = *.02), and physical activity attitudes (*P* = .05) (Table 1).

**Figure.**
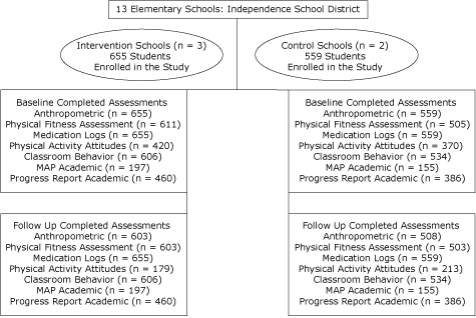
Study flow chart, ABC (Activity Bursts in the Classroom) for Fitness Program.MAP indicates Missouri Academic Performance test.

**Table:** 

This flow chart depicts a series of circles and boxes that describes the response rates for the ABC for Fitness Program. The first box is arranged horizontally and says, “13 Elementary Schools: Independence School District.” Two ovals are beneath this box and are beside each other. A line connects the first box to 4 boxes. The 2 ovals are directly beneath the first box and are divided by the line. The first oval reads, “Intervention schools (n = 3) (655 Students Enrolled in the Study). The second oval says, “Control Schools (N = 2), (559 Students Enrolled in the Study). Beneath the ovals are 2 boxes arranged horizontally.
Directly beneath the first oval is a box. The first box says, “Baseline Completed Assessments, Anthropometric (n = 655); Physical Fitness Assessment (n = 611), Medication Logs (n = 655); Physical Activity Attitudes (n = 420), Classroom Behavior (n = 606) MAP Academic (n = 197); Progress Report Academic (n = 460).”
To the right of the first box, the second box says, “Baseline Completed Assessments, Anthropometric (n = 559); Physical Fitness Assessment (n = 505), Medication Logs (n = 559); Physical Activity Attitudes (n = 370), Classroom Behavior (n = 534), MAP Academic (n = 155); Progress Report Academic (n = 386).”
Beneath the first box is a third box, which is connected to the first box by a line. The third box says, “Follow Up Completed Assessments, Anthropometric (n = 603); Physical Fitness Assessment (n = 603), Medication Logs (n = 655); Physical Activity Attitudes (n = 179), Classroom Behavior (n = 606), MAP Academic (n = 197); Progress Report Academic (n = 460).”
The fourth box is to the right of the third box; a line connects it to the second box. The fourth box reads, “Follow Up Completed Assessments Anthropometric (n = 508); Physical Fitness Assessment (n = 503), Medical Logs (n = 559); Physical Activity Attitudes (n = 213), Classroom Behavior (n = 534), MAP Academic (n = 155); Progress Report Academic (n = 386).”

Improvements in anthropometric measures at follow-up in the control group were found to be significant compared with those of the intervention group (weight [*P* = .01] and BMI [*P*  = .02]) (Table 2). The intervention group showed greater improvement in physical fitness than did the control group on measures of abdominal strength (*P* <.001), upper-body strength (*P* <.001), and trunk extensor (*P* <.001). Greater improvement was observed in the control group compared with the intervention group in right-side flexibility (*P* = .03) (Table 2). In logistic regression analysis, school was a predictor of aerobic capacity (*P* <.001), abdominal strength (*P*  <.001), upper-body strength (*P* = .05), and trunk extensor (*P* <.001); however, it was not found to be a predictor of left-side (*P* = .20) or right-side flexibility (*P* = .47). School was also a predictor of anthropometric measures (*P* = .03), use of medication (*P* = .01), mathematics performance (*P* = .004), and communication arts performance (*P* = .02).

Students in the intervention group showed significantly reduced use of asthma medication compared with students in the control group (*P* = .03). Students in the intervention group also showed reduced use of ADHD medication compared with students in the control group (*P* = .07). The intervention group had a greater reduction in asthma medication use and ADHD medication use combined at follow-up compared with the control group (*P* = .005) (Table 2).

No significant differences were seen between the intervention and control groups in behavior changes from baseline as measured by the ISD Work/Social Skills progress report (*P* = .86) (Table 2). No significant differences between groups were seen in attitudes toward physical activity (*P* > .05).

Academic performance measured by MAP achievement level scores showed no significant differences between the intervention and control groups in reading (*P *= .35) and mathematics (*P* = .15). However, as measured by ISD progress reports, significant differences in academic performance were observed. In mathematics, the control group had a greater proportion of students whose academic performance improved compared to that of the intervention group (28.6% vs 20.8%, *P* <.001). The proportion of students in the control group whose reading level improved was also greater than that of the intervention group (21.1% vs 16.1%, *P* = .01).

## Discussion

This initial controlled study of ABC for Fitness suggests that the program can improve fitness, reduce medication use, and preserve teaching time and academic performance. The program can be used in elementary school classrooms with minimal interruptions in daily classroom management related to academics and classroom behavior. We found that students in the intervention group improved significantly in physical fitness and in reduced use of ADHD and asthma medication compared with the control group. Few differences were observed between the intervention and control schools in terms of physical activity attitudes, academic performance, and classroom behavior; however, measures such as these are less likely to show improvement in the short term. This study successfully influenced some of the "upstream" factors that over time can influence measures of weight and overall health.

Several studies have shown how classroom-based physical activity programs can effectively improve behavior and increase in-school physical activity and overall physical activity (20,21). Our study also demonstrates the efficacy of classroom-based physical activity without any change in basic curriculum. This distinction is meaningful because no extra time needs to be devoted to the program as a result of increased classroom time efficiency. Focus groups showed acceptance by students, teachers, parents, and administrators. Even the best programs will not work if they are not accepted and implemented enthusiastically. The compatibility of the ABC for Fitness program with the prevailing needs and priorities of schools is suggested by the widespread dissemination of the program by word-of-mouth alone; to our knowledge, hundreds and perhaps thousands of US schools are using the program (13).

Previous programs have attempted to add a new section into the curriculum, which forces the reduction or elimination of other educational time. The ABC for Fitness program can reduce downtime and increase teaching time. As several schools decrease the amount of time and money spent on physical education (22), this program is an alternative; training time is minimal, and no specialists or extra equipment is required.

This intervention was not projected to change anthropometric measures in the short term, although that is a goal. The program may take years to influence health outcomes; meanwhile, other factors such as dietary patterns and social environments will need to be addressed. A more in-depth intervention, performed over a longer period, would allow these outcomes to be elucidated. As with ABC for Fitness, other health promotion programming can be targeted to the existing school calendar and meet these goals without interfering with the primary mission of schools (23).

This study showed improvements in measures of fitness during 1 school year. Childhood physical activity has been shown to have a positive correlation with well-being in adulthood (24-26), in part because of the development and maintenance of good habits. A recent study has also concluded that physical activity may be positively correlated with academic achievement (2). Studies have suggested that physical activity can decrease the symptoms of ADHD (27,28) and asthma (29-32). Our program echoed these findings by showing a significant decrease in medication use for asthma and a decrease in ADHD medication.

One concern of adding a new curriculum is the effect on behavior and academic performance, especially if the program involves physical activity. The program was designed with the intent to replace the time that teachers use to settle students down with structured activity bursts. By designing the program in this manner, it was believed that the time lost with the ABC program would not worsen academic performance or classroom behavior; this appears to be the case, because no significant change was seen in classroom behavior or MAP scores. Several studies have suggested the potential for physical activity to improve academics (2,33,34).

BMI decreased significantly in the control group compared with the intervention group. Most studies that have evaluated the effects of physical activity on BMI in elementary schools have found no beneficial effect (35). It is unclear why in this study, the control group was associated with reduced BMI.

Our study had several limitations. First, our population was not a true cross-section of all children; ISD is just 1 school district in the midwestern United States. The ISD population is predominantly white and has a high proportion of low-income students. The sample was small. Only 1 school district was used for the study, which made it difficult to assess academic performance and develop a large enough power to assess all outcome measures. The periods of intervention and follow-up were limited.

We plan a larger-scale study based on the results of this study that will entail a larger cohort and multiple school districts for a more accurate cross-section of US children, better teacher training, and longer follow-up to allow ample time to see results. Additionally, we plan to combine ABC for Fitness with other intervention components, including nutritional education to children and parents, to influence downstream measures.

This study demonstrates the feasibility of bursts of structured physical activity for elementary school students for 30 minutes or more of daily physical activity without reducing teaching time or requiring any special facilities. Beneficial effects on daily physical activity, fitness, and measures of health are suggested. The program can easily be incorporated into almost any school routine. Thus, we recommend concurrent replication and dissemination and more intensive study of the program.

## Figures and Tables

**Table 1:** Characteristics of Elementary School Children in the ABC for Fitness Program, Independence, Missouri, 2007-2008

Characteristic	Intervention (n = 655)	Control (n = 559)	*P* Value
**Sex, n (%)**			
Boys	326 (49.8)	267 (47.8)	.49
Girls	329 (50.2)	292 (52.2)	
**Grade level, n (%)**			
Second	219 (33.4)	184 (32.9)	.98
Third	217 (33.1)	186 (33.3)	
Fourth	219 (33.4)	189 (33.8)	
**Age, n (%)**			
7 y	168 (26.0)	143 (25.7)	.89
8 y	214 (33.0)	176 (31.6)	
≥9 y	273 (41.1)	238 (42.7)	
**Anthropometric measures, median (IQR)**			
Weight, lbs	67.0 (58.0-82.0)	63.0 (55.0-78.0)	<.01
BMI, kg/m²	17.7 (16.1-0.4)	17.3 (15.8-19.9)	.06
BMI percentile	74.0 (50.0-91.0)	71.0 (44.0-90.0)	.21
**Physical fitness, mean (SD)**			
Aerobic capacity, VO_2max_ a	46.0 (44.0-48.0)	46.2 (44.8-49.2)	<.01
Abdominal strength (curl-ups)	13.0 (5.0-23.0)	17.0 (8.0-25.0)	
Upper-body strength (90-degree push-ups)	6.0 (2.0-12.8)	13.0 (9.0-15.0)	
Trunk extensor (trunk lifts)	7.0 (6.0-9.0)	8.0 (7.0-9.5)	
Left-side flexibility (back-saver sit and reach)	10.0 (8.0-11.0)	11.0 (8.5-23.0)	
Right-side flexibility (back-saver sit and reach)	10.0 (8.5-11.0)	11.5 (9.0-24.0)	
**Medication use, n (%)**			
ADHD	21 (3.2)	14 (2.5)	.47
Asthma	37 (5.6)	26 (4.7)	.44
Either medication	58 (8.8)	40 (7.2)	.29
**Physical activity attitudes, mean (SD)**			
Physical activity survey total score	9.3 (7.2-11.4)	8.9 (6.7-11.1)	.05
**Classroom behavior, mean (SD)**			
Progress report: work/social skills mean score	2.7 (2.3-3.1)	2.8 (2.4-3.2)	.02
**2007 MAP scores for third-grade students, %**			
**Mathematics achievement level **			
Below basic	4.0	1.9	.14
Basic	36.4	32.9	
Proficient	39.9	51.0	
Advanced	19.7	14.2	
**Communication arts achievement level**			
Below basic	5.6	4.5	.22
Basic	43.4	37.4	
Proficient	27.6	38.1	
Advanced	23.5	20.0	
**2007-2008 ISD progress report scores, %**			
**Mathematics: numbers operations**			
Below basic	4.1	4.6	.08
Basic	34.0	42.6	
Proficient	59.3	51.4	
Advanced	2.6	1.4	
**Communication arts: reading level **			
Below basic	8.2	8.0	.48
Basic	22.3	22.9	
Proficient	38.5	42.6	
Advanced	31.0	26.5	

Abbreviations: ABC, Activity Bursts in the Classroom; IQR, interquartile range; BMI, body mass index; ADHD, attention-deficit hyperactivity disorder; MAP, Missouri Academic Performance; ISD, Independence School District.

a VO_2max _is maximal oxygen consumption, a proxy for general fitness.

**Table 2:** Changes from Baseline for Participants in the ABC for Fitness Program, Independence, Missouri, 2007-2008

Characteristics	Intervention Group	Control Group	*P* Value
**Anthropometric measures, median (IQR)**			
Weight, lb	5.0 (2.0 to 7.0)	4.0 (1.0 to 7.0)	.01
BMI, kg/m²	0.3 (−0.2 to 0.8)	0.1 (−0.3 to 0.7)	.02
BMI percentile	0 (−4.0 to 2.3)	−1.0 (−6.0 to 2.0)	.07
**Physical fitness, median (IQR)**			
Aerobic capacity, VO_2max_ a	−1.0 (−1.2 to 1.2)	−1.0 (−1.2 to 1.3)	.02
Abdominal strength (curl-ups)	9.0 (0.0 to 28.5)	0.0 (−4.0 to 7.0)	<.001
Upper-body strength (90-degree push-ups)	2.0 (0.0 to 6.0)	0.0 (−3.5 to 2.0)	<.001
Trunk extensor (trunk lifts)	1.0 (0.0 to 3.0)	1.0 (0.0 to 2.0)	<.001
Left-side flexibility (back-saver sit and reach)	0.0 (−1.0 to 0.5)	0.0 (−1.0 to 0.5)	.33
Right-side flexibility (back-saver sit and reach)	0.0 (−0.05 to 1.0)	0.0 (−0.5 to 1.0)	.03
**Medication use, n (%)**			
ADHD (yes changed to no)	7 (33.3)	1 (7.1)	.07
ADHD (no changed to yes)	6 (0.9)	4 (0.7)	.70
Asthma (yes changed to no)	5 (16.7)	0 (0)	.03
Asthma (no changed to yes)	0 (0.0)	1 (0.2)	.35
Either medication (yes changed to no)	12 (23.5)	1 (2.6)	.005
Either medication (no changed to yes)	6 (1.0)	4 (0.8)	.68
**Physical activity attitudes, mean (SD)**			
Physical activity survey total score	0.02 (−2.14 to 2.18)	0.19 (−1.83 to 2.21)	.41
**Classroom behavior, mean (SD)**			
Progress report: work/social skills	0.04 (−0.21 to 0.29)	0.05 (−0.21 to 0.31)	.86

Abbreviations: ABC, Activity Bursts in the Classroom; IQR, interquartile range; BMI, body mass index; ADHD, attention-deficit hyperactivity disorder.

a VO_2max _is maximal oxygen consumption, a proxy for general fitness.
